# Co-circulation of dengue, chikungunya, and Zika viruses in Colombia from 2008 to 2018

**DOI:** 10.26633/RPSP.2019.49

**Published:** 2019-06-07

**Authors:** Alejandro Rico-Mendoza, Porras-Ramírez Alexandra, Aileen Chang, Liliana Encinales, Rebecca Lynch

**Affiliations:** 1 Grupo de Medicina Comunitaria y Salud Colectiva Grupo de Medicina Comunitaria y Salud Colectiva Universidad El Bosque Bogotá Colombia Grupo de Medicina Comunitaria y Salud Colectiva, Universidad El Bosque, Bogotá, Colombia.; 2 Department of Medicine, the George Washington University Department of Medicine, the George Washington University WashingtonD.C United States of America Department of Medicine, the George Washington University, Washington, D.C., United States of America.; 3 Allied Research Society Allied Research Society BarranquillaAtlántico Colombia Allied Research Society, Barranquilla, Atlántico, Colombia.; 4 Department of Microbiology, Immunology, and Tropical Medicine, the George Washington University Department of Microbiology, Immunology, and Tropical Medicine, the George Washington University WashingtonD.C United States of America Department of Microbiology, Immunology, and Tropical Medicine, the George Washington University, Washington, D.C., United States of America.

**Keywords:** Zika virus, dengue virus, chikungunya virus, coinfection, Colombia, Virus Zika, virus del dengue, virus chikungunya, coinfección; Colombia, Zika virus, vírus da dengue, vírus chikungunya, coinfecção, Colômbia

## Abstract

**Objective.:**

This study aimed to identify the co-circulation patterns of three viruses (dengue, Zika, and ­chikungunya) in Colombia from 2008 to 2018 by using notification reports provided to the national surveillance system.

**Methods.:**

This cross-sectional study was conducted through a review of data for 2008 through 2018 from Colombia’s Public Health Surveillance System (SIVIGILA).

**Results.:**

In 2015, when chikungunya was first detected, it had a higher incidence (1 359.0 cases per 100 000 persons) than did the two other diseases. In 2016, when the circulation of Zika virus was first found, the incidence was 296.4 cases per 100 000 persons; that incidence declined dramatically in the next two years. Between 2015 and 2018, there was a substantial decrease in the frequency of dengue circulation, with it going from 334.1 cases per 100 000 persons in 2015 to 90.7 cases per 100 000 in 2017 and 173.1 cases per 100 000 in 2018.

**Conclusions.:**

The decrease in the number of dengue cases after co-circulation of the three viruses could indicate possible cross-protection. This finding should be further analyzed.

## RESUMEN

**Objetivo:**

Establecer las características de la cocirculación de tres virus (dengue, Zika y chikunguña) en Colombia desde el 2008 hasta el 2018. Para ello, en este estudio se han utilizado los informes de notificación que se proporcionan al sistema nacional de vigilancia.

**Métodos:**

Este estudio transversal se llevó a cabo mediante el análisis de los datos correspondientes al período 2008-2018 del Sistema Nacional de Vigilancia en Salud Pública de Colombia (SIVIGILA).

**Resultados:**

En el 2015, cuando se detectó por primera vez el chikunguña, este virus tuvo una incidencia mayor (1 359,0 casos por 100 000 personas) que las otras dos enfermedades. En el 2016, cuando se detectó por primera vez la circulación del virus del Zika, la incidencia fue de 296,4 casos por 100 000 personas; el número de casos disminuyó enormemente en los siguientes dos años. Entre el 2015 y el 2018, se observó una reducción sustancial en la frecuencia de la circulación del dengue: se pasó de 334,1 casos por 100 000 personas en el 2015 a 90,7 casos por 100 000 en el 2017 y a 173,1 casos por 100 000 en el 2018.

**Conclusiones:**

La disminución en el número de casos del dengue que siguió a la cocirculación de los tres virus podría indicar una posible protección cruzada. Este resultado debe analizarse en otros estudios.

## RESUMO

**Objetivo.:**

Identificar padrões de co-circulação de três vírus (dengue, Zika e chikungunya) na Colômbia de 2008 a 2018 mediante análise de casos notificados ao sistema nacional de vigilância.

**Métodos.:**

Estudo transversal realizado através de análise de dados de 2008 a 2018 obtidos do Sistema de Vigilância em Saúde Pública da Colômbia (SIVIGILA).

**Resultados.:**

Em 2015, quando o chikungunya foi detectado pela primeira vez, sua incidência foi superior à das duas outras doenças (1.359,0 casos por 100,000 habitantes). Em 2016, quando a circulação do vírus zika foi detectada pela primeira vez, a incidência foi de 296,4 casos por 100,000 pessoas, decaindo drasticamente nos dois anos seguintes. Entre 2015 e 2018, houve uma redução importante na frequência de circulação do vírus dengue, de 334,1 casos por 100,000 habitantes em 2015 a 90,7 casos por 100,000 em 2017 e 173,1 casos por 100,000 em 2018.

**Conclusões.:**

A redução do número de casos de dengue após o estabelecimento da co-circulação dos três vírus pode indicar proteção cruzada. Este achado requer análise adicional.

In Latin America and the Caribbean, the recent epidemics of mosquito-borne arboviruses, including the chikungunya virus (CHIKV) and Zika virus (ZIKV), combined with the preexistence of the dengue virus (DENV) (1, 2), have resulted in the circulation of three substantially pathogenic arboviruses that exhibit similar acute symptoms (3-6).

In the Americas, CHIKV, which is an alphavirus, emerged for the first time at the end of 2013, and has infected over one million individuals since then (5). ZIKV is a flavivirus that emerged in the Americas in 2015 and 2016. An estimated 80% of acute ZIKV infections are asymptomatic, and the remaining 20% ​​clinically resemble CHIKV and DENV infection, including with symptoms of fever, rash, headache, and arthralgia (5-7). Neurological complications, including  Guillain–Barré syndrome, have also been reported after ZIKV infection (8, 9). Importantly, ZIKV infection during pregnancy is associated with severe teratogenic effects, including microcephaly (8-11).

Co-circulation not only makes differential diagnoses more complicated but also leads to poorly characterized disease manifestations during viral coinfection (12-15).

This study aims to describe the co-circulation of DENV, CHIKV, and ZIKV in Colombia, in order to identify epidemiological patterns that may provide insight into the immunologic interactions of these co-circulating viral infections in that country.

## MATERIALS AND METHODS

This cross-sectional study was performed in Colombia by reviewing data from the country’s Public Health Surveillance System, which is under the leadership of the country’s National Institute of Health.

### Study area

Located in northwest South America, Colombia has a population of 47 million people. It is divided administratively and politically into 33 divisions: 32 departments (with their respective capital cities) and the capital district, Bogotá. The departments of Antioquia, Boyacá, Caldas, Cauca, Cundinamarca, Huila, Nariño, Norte de Santander, Quindío, Risaralda, Santander, Tolima, and Valle del Cauca compose the Colombian Andes, and most of their capital cities are at an altitude of more than 2 000 m above sea level. The departments of Boyacá, Cundinamarca, and Nariño have a cold climate, but the departments of Antioquia, Caldas, Cauca, Norte de Santander, Quindío, Santander, and Tolima are in a temperate or warm climate. The departments of Huila, Risaralda, and Valle del Cauca are in warm climates.

### Colombian public health surveillance system

Colombia’s Public Health Surveillance System (SIVIGILA) consists of an organized association of users, rules, procedures, and resources (financial, technical, and human) for the collection of data and for the analysis, interpretation, and dissemination of information regarding health events. Among the health concerns that SIVIGILA monitors are dengue, Zika, and chikungunya. Information on these diseases flows from clinics and hospitals to organizations responsible for health insurance and to territorial health entities, where data is consolidated and then sent to the National Institute of Health (INS), which is the governing body for health surveillance in Colombia.

### Data collection and case definitions

The data reported in this article correspond to the cases reported to SIVIGILA. As described below, three SIVIGILA case definitions were used for data collection.

Dengue cases were defined as all people with acute febrile illness (< 7 days) with two or more of the following manifestations: headache, retro-orbital pain, myalgia, arthralgia, or rash. Dengue cases of concern included anyone who met the above definition and also displayed any of the following warning signs: intense pain, continuous abdominal pain, persistent vomiting, diarrhea, drowsiness and/or irritability, postural hypotension, painful hepatomegaly greater than 2 cm, decreased diuresis, hypothermia, mucous membrane hemorrhage, or abrupt drop in platelet levels (< 100 000) associated with hemoconcentration.

Zika cases were defined as all people with laboratory- confirmed natural circulation of ZIKV two weeks before the onset of symptoms and who presented with rash and one or more of the following signs: fever < 38.5 °C, nonpurulent conjunctivitis or conjunctival hyperemia, arthralgia, myalgia, headache, or general discomfort. Laboratory confirmation included the detection of Zika-specific IgM antibodies in the serum.

Chikungunya cases were defined as all people who presented with fever > 38 °C, severe arthralgia or acute onset arthritis, erythema multiform, or symptoms that were not explained by other medical conditions. Furthermore, individuals must have resided or visited a municipality with evidence of CHIKV circulation or a municipality within 30 km of a municipality with viral circulation.

### Data analysis and data summary

A descriptive data analysis involving the absolute and relative frequencies of variables and their proportions by department was performed. Additionally, the 95% confidence intervals (95% CIs) of the relative frequencies were determined. Quantitative variables, such as age, were described using median, standard deviation, minimum, and maximum values. Incidence was calculated per 100 000 inhabitants, and frequencies and percentages were used to describe the main characteristics of the cases for variables such as age, sex, and department. The rates were also stratified by department.

### Study period

For dengue, data on cases reported since 2008 were used in this study. However, in addition, cases were reviewed since the 1970s to understand the historical behavior of DENV circulation in Colombia. For CHIKV and ZIKV, data obtained since these viruses first circulated in the country were used.

### Data quality check

Because the information used in this study originated from secondary sources, misclassification bias was a potential limitation. We attempted to minimize this bias by using only laboratory-confirmed cases that were reported to the SIVIGILA.

### Geographic distribution of cases

This analysis aimed to describe the geographic distribution of Zika, dengue, and chikungunya cases in Colombia based on the residence of people diagnosed with these viruses, and to identify areas with a high incidence of cases and co-circulation of the three viruses.

Colombia’s 1991 constitution institutes the country as a unitary republic that is divided administratively and politically into 33 divisions: 32 departments and a capital district, Bogotá. The departments form geographic, cultural, and economic regions. In Colombia, resources pass from the nation to the departments and from departments to the municipalities, except for Bogotá, which receives resources directly from the nation because it is the capital district.

Besides the 32 departments and the capital district, Colombia also has special districts and metropolitan areas. The special districts are municipalities that stand out for aspects such as their economic, political, or population weight (1), and the metropolitan areas correspond to the subregional integration of departmental capitals. Colombia has 1 101 registered municipalities (including five special districts), plus 20 nonmunicipalized areas and the island of San Andrés.

### Ethical aspects

Because data from anonymous secondary SIVIGILA sources was used, this study was classified as without risk, according to the current ethical norms in Colombia. This classification includes studies that employ techniques and methods of retrospective documentary research and those in which no intervention or intentional modification of biological, physiological, psychological, or social factors of the individuals participating in the study is performed, which includes medical record reviews, interviews, questionnaires, and other methods by which patients could be identified or sensitive aspects of their behavior could be revealed.

## RESULTS

### Dengue virus

In 2016, 101 016 dengue cases were reported to SIVIGILA, of which 59 114 had no warning signs, 41 003 displayed warning signs, and 899 were severe dengue. Guainía, Casanare, and Nariño departments had the highest proportions of DENV cases without warning signs; Cesar, La Guajira, and Santa Marta (­district) had the highest proportions with warning signs. Of the dengue cases reported, 41 690 (41.3%; 95% CI: 40.9%–41.5%) were laboratory confirmed, 7 022 (7%; 95% CI: 6.7%–7.1%) were confirmed by epidemiological linkage, and 52 304 (51.8%; 95% CI: 51.2%–51.8%) were suspected cases. For severe dengue cases, 759 were laboratory confirmed (84.4%; 95% CI: 81.9%–86.6%). Of the DENV cases, 50% (95% CI: 49.6%–50.3%) occurred in men, with young children particularly affected. For example, 24.8% (95% CI: 23.9%–25.1%) of total DENV cases and 35.5% (95% CI: 34.9%–35.8%) of severe DENV cases were reported in children less than 15 years of age. Moreover, 884 cases were reported in the indigenous population (0.9%; 95% CI: 0.81%–0.93%) and 2 186 in Afro-Colombians (2.2%; 95% CI: 2.07%–2.25%).

Of the DENV cases, 84.5% (95% CI: 84.0%–85.1%) were reported from 10 Colombian departments: Antioquia (27.6%; 95% CI: 27.0%–28.2%), Valle del Cauca (25.4%; 95% CI: 25.0%–25.9%), Santander (6.9%; 95% CI: 6.7%–7.1%), Tolima (5.7%; 95% CI: 5.3%–6.1%), Cundinamarca (4.4%; 95% CI: 4.1%–5.0%), Huila (3.9%; 95% CI: 3.2%–4.1%), Risaralda (2.9%; 95% CI: 2.1%–3.1%), Norte de Santander (2.7%; 95% CI: 2.3%–3.0%), Meta (2.6%; 95% CI: 2.1%–3.0%), and Quindío (2.5%; 95% CI: 2.0%–2.8%). For severe dengue, 80.3% (95% CI: 80.0%–81.2%) of the cases were distributed as follows: Valle del Cauca (22.2%; 95% CI: 21.9%–23.0%), Tolima (13.6%; 95% CI: 13.0%–14.2%), Huila (12.5%; 95% CI: 12.1%–13.1%), Antioquia (12.3%; 95% CI: 12.1%–12.5%), Santander (7.7%; 95% CI: 7.3%–8.1%), Atlántico (3.9%; 95% CI: 3.7%–4.1%), Meta (3.1%; 95% CI: 3.0%–3.5%), Cundinamarca (2.8%; 95% CI: 2.5%–3.1%), and Norte de Santander (2.2%; 95% CI: 2.1%–2.6%).

In 2017, 26 279 cases were reported in total, including 15 369 (58.5%; 95% CI: 57.8%–59.0%) without warning signs, 10 624 (40.4%; 95% CI: 39.8%–41.0%) with warning signs, and 286 (1.1%; 95% CI: 0.9%–1.2%) severe dengue cases. Additionally, 57.4% (95% CI: 56.4%–57.6%) of DENV cases occurred in men. Again, DENV had a special effect on young children, with 12.5% (95% CI: 12.0%–13.1%) ​​of total DENV cases and 15.4% (95% CI: 14.9%–15.9%) of severe DENV cases reported in children under 5 years of age. Furthermore, 298 cases were reported in the indigenous population (1.1%; 95% CI: 1.0%–1.2%) and 838 in Afro-Colombians (3.2%; 95% CI: 2.9%–3.4%). There were 198 cases of infection in pregnant women (1.8%; 95% CI: 1.6%–2.3%). Of the dengue cases with warning signs, 63.7% (95% CI: 62.5%–64.5%) were hospitalized in 2017, and 21 deaths from DENV were confirmed.

Through epidemiological week 4 of 2018, 2 182 DENV cases were reported, including 1 114 (51.0%; 95% CI: 48.9%–53.1%) without warning signs, 1 031 (47.7%; 95% CI: 45.1%–49.3%) with warning signs, and 37 (1.7%; 95% CI: 1.2%–2.3%) with severe dengue. Additionally, eight deaths due to DENV were reported.

### Chikungunya virus

In Colombia, the first laboratory-confirmed autochthonous cases of CHIKV were reported in September 2014. These cases occurred in people from rural areas in Bolívar department. From 2014 to 2016, 19 435 CHIKV cases were reported in Colombia. Of these cases, 202 (1.03%; 95% CI: 0.9%–1.1%) were laboratory confirmed, 19 003 (97.7%; 95% CI: 97.5%–97.9%) displayed clinical criteria, and 230 were suspected cases (1.18%; 95% CI: 1.03%–1.34%). On average, 405 cases were reported each week in 2016. During the epidemic, CHIKV circulation was confirmed via virological testing in 758 municipalities and 4 districts of the country.

Of the reported CHIKV cases, 63.4% (95% CI: 63.1%–64.0%) occurred in women, and 11.1% (95% CI: 10.9%–11.5%) were reported in those 25 to 29 years old. The municipalities with the largest share of cases (percentage of total cases reported) were Cali (15.9%; 95% CI: 15.3%–16.1%), Bucaramanga (5.1%; 95% CI: 5.0%–5.6%), Ibagué (4.5%; 95% CI: 4.0%–5.1%), Pereira (3.9%; 95% CI: 3.8%–4.1%), Barranquilla (3.6%; 95% CI: 3.4%–3.9%), Villavicencio (3.2%; 95% CI: 3.0%–3.9%), Dosquebradas (3.02%; 95% CI: 3.00%–3.51%), Floridablanca (2.5%; 95% CI: 2.3%–2.9%), Florencia (1.6%; 95% CI: 1.5%–1.8%), and San Andrés de Sotavento (1.6%; 95% CI: 1.4%–2.1%).

In 2016, the national incidence of CHIKV in urban populations was 72.4 cases per 100 000 inhabitants. In addition, 12 deaths associated with CHIKV infection were reported.

In 2017, SIVIGILA was notified of 1 128 potential chikungunya cases, of which 34 (3.0%; 95% CI: 2.1%–4.1%) were laboratory confirmed, 1 050 (93.1%; 95% CI: 91.4%–94.4%) were clinically confirmed, and 44 (3.9%; 95% CI: 2.8%–5.1%) were suspected cases. Also in 2017, the national incidence of chikungunya in urban populations was 4.0 cases per 100 000 inhabitants. The five regional entities with the highest incidence were Guaviare, Putumayo, Cundinamarca, Casanare, and Tolima.

Through epidemiological week 4 of 2018, 55 cases  were reported, of which 52 (94.5%; 95% CI: 85.8%–98.5%) were clinically confirmed and 3 (5.4%; 95% CI: 1.4%–14.1%) were suspected.

### Zika virus

From 9 August 2015 through 2 April 2016, a total of 65 726 cases of ZIKV were reported in Colombia, with 2 485 (4%) that were positive on RT-PCR assay. Among the 65 726 patients who were reported to have ZIKV, 2 336 (4%) were hospitalized at the time that the case was reported, including 938 (8%) of the 11 944 pregnant women. The number of reported ZIKV cases steadily increased from October 2015 through January 2016, with the largest number of cases being reported during the week of 31 January to 6 February (epidemiologic week 5)

In 2017, 1 750 ZIKV cases were reported, of which 57 were laboratory confirmed. The departments with the most ZIKV cases were Valle del Cauca, Santander, Tolima, Cundinamarca, and Meta, representing 65.7% (95% CI: 65.0%–66.1%) of all cases. An analysis by age and gender showed that 55.4% (95% CI: 55.0%–56.1%) of cases occurred in women; 27.9% (95% CI: 24.4%–31.6%) occurred in children younger than 1 year; 13.3% (95% CI: 13.0%–14.1%) occurred in those 25 to 29 years old; and 12.7% (95% CI: 12.1%–13.4%) occurred in persons 20 to 24 years old. Since the beginning of 2017, 388 cases have been identified in pregnant women who reported having symptoms compatible with ZIKV infection. Pregnant women represented 22.1% (95% CI: 20.2-24.1%) of all ZIKV cases, and 38 (14.3%; 95% CI: 13.9%–15.1%) of the cases in pregnant women were laboratory confirmed.

In 2015 and 2016, of the cases exhibiting neurological syndromes associated with ZIKV (Guillain–Barré syndrome, ascending polyneuropathies, or other similar neurological conditions), 270 cases with a history of febrile illness compatible with ZIKV infection were reported. In 2017, only 77 cases were reported, which were more frequent in the group of 10 to 19 years (Table 1). Of these 77 cases, 72 (93.5%; 95% CI: 93.0%–94.2%) were hospitalized, and 3 died. Additionally, 45 of the cases (58.4%; 95% CI: 47.2%–69.0%) occurred in men.

**TABLE 1:** Number and percentage (with 95% confidence interval (CI)) of Zika cases with neurological syndrome by age group and sex in Colombia, 2017

Age group (yr)	Females			Males			Total		
Number	%	95% CI	Number	%	95% CI	Number	%	95% CI
< 1	0	0.0	0.0–0.0	0	0.0	0.0–0.0	0	0.0	0.0–0.0
1–4	0	0.0	0.0–0.0	1	1.3	0.06–6.2	1	1.3	0.06–6.2
5–9	1	1.3	0.06–6.2	1	1.3	0.06–6.2	2	2.6	0.4–8.3
10–14	3	3.9	1.0–10.2	10	13.0	6.7–21.9	13	16.9	9.7–26.4
15–19	7	9.1	4.0–17.1	3	3.9	1.0–10.2	10	13.0	6.7–21.9
20–24	1	1.3	0.06–6.2	2	2.6	0.4–8.3	3	3.9	1.0–10.2
25–29	3	3.9	1.0–10.2	0	0.0	0.0–0.0	3	3.9	1.0–10.2
30–34	4	5.2	1.6–12.0	5	6.5	2.4–13.8	9	11.7	5.8–20.3
35–39	5	6.5	2.4–13.8	3	3.9	1.0–10.2	8	10.4	4.9–18.7
40–44	3	3.9	1.0–10.2	5	6.5	2.4–13.8	8	10.4	4.9–18.7
45–49	0	0.0	0.0–0.0	3	3.9	1.0–10.2	3	3.9	1.0–10.2
50–54	0	0.0	0.0–0.0	3	3.9	1.0–10.2	3	3.9	1.0–10.2
55–59	3	3.9	1.0–10.2	1	1.3	0.06–6.2	4	5.2	1.6–12.0
60–64	1	1.3	0.06–6.2	3	3.9	1.0–10.2	4	5.2	1.6–12.0
≥ 65	1	1.3	0.06–6.2	5	6.5	2.4–13.8	6	7.8	3.2–15.5
Total	32	41.6	30.9–52.7	45	58.4	47.2–69.0	77	100.0	96.1–100.0

***Source:*** Data were obtained from Colombia’s Public Health Surveillance System (SIVIGILA), and then prepared for this article by the authors.

## Co-circulation of DENV, CHIKV, and ZIKV in Colombia

**FIGURE 1:**
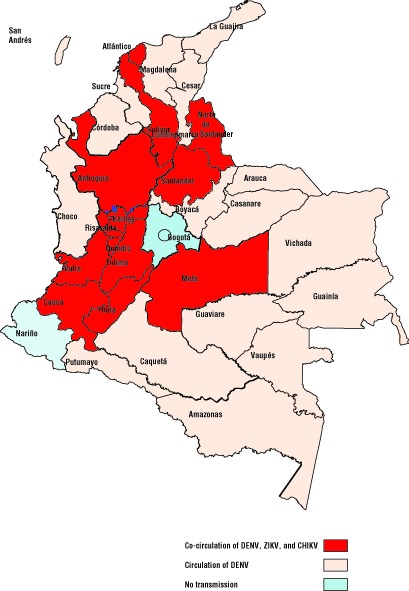
Departments of Colombia with co-circulation of dengue (DENV), Zika (ZIKV), and chikungunya (CHIKV), 2015 to 2018

Colombia is a hyperendemic country for DENV transmission (1). In 2014, the situation was further complicated with CHIKV circulation (2), followed by ZIKV circulation in 2015 (3). This combination of similar viruses circulating in the country presents challenges regarding case confirmation, given the similar clinical presentations and cross-reactivity of DENV and ZIKV in serologic tests. As shown in Table 2, two or all three arboviral diseases have co-circulated in Colombia since 2015, with the highest incidence generally reported for dengue. In 2015, CHIKV had the highest incidence of the three viruses, whereas by 2016, the rate of ZIKV had increased and dengue circulation had decreased compared with historical averages. The following departments reported co-circulation of the three viruses: Antioquia, Atlántico, Bolívar, Caldas, Cauca, Huila, Meta, Norte de Santander, Quindío, Risaralda, Santander, Tolima, and Valle del Cauca (Figure 1).

**TABLE 2:** Incidence of dengue (DENV), chikungunya (CHIKV), and Zika (ZIKV), per 100 000 individuals in Colombia, from 2008 to 2018

Year	DENV	CHIKV	ZIKV
2008	161.6	0.0	0.0
2009	225.2	0.0	0.0
2010	664.5	0.0	0.0
2011	128.1	0.0	0.0
2012	221.7	0.0	0.0
2013	474.6	0.0	0.0
2014	403.6	0.0	0.0
2015	344.1	1 359.0	0.0
2016	356.3	72.4	296.4
2017	90.7	3.5	6.3
2018	173.1	2.5	3.3

***Source:*** Data were obtained from Colombia’s Public Health Surveillance System (SIVIGILA), and then prepared for this article by the authors.

## DISCUSSION

DENV, CHIKV, and ZIKV are arboviruses of great concern because of their impact on public health, particularly in countries such as Colombia. Based on DENV endemicity patterns, transmission patterns of these newer arboviruses can be determined. DENV has been the most prevalent arbovirus in Colombia for the last several decades. DENV is a public health priority in Colombia for multiple reasons. Its reemergence and intense transmission, with an increasing tendency toward frequent and severe DENV outbreaks, are particularly concerning. The simultaneous circulation of different serotypes, reintroduction of serotype 3, and infestation by *Aedes aegypti* in more than 90% of the country’s territory located at less than 2 200 m above sea level make DENV circulation challenging to control.

Furthermore, the introduction of *Aedes albopictus* and the growing trend of urbanization of the Colombian population because of recent violent conflicts in multiple areas further complicate dengue control. Finally, DENV tends to erupt in epidemic cycles every two to three years, but these cycles can be challenging to predict. For example, the epidemics of 1977, 2002, 2007, and 2010 were notable for the high numbers of cases. The 2010 outbreak is considered the most massive registered DENV epidemic in Colombia, with more than 150 000 confirmed cases, 217 deaths, and simultaneous circulation of all four serotypes. Interestingly, the number of DENV cases reported in 2015 decreased during the large-scale CHIKV and ZIKV epidemics. Various possibilities might explain this phenomenon. For example, there may have been underreporting of cases during the Zika epidemic because the only institution in Colombia that could do laboratory confirmation was the INS. Another possibility is that mosquitoes are unable to transport and transmit DENV simultaneously with CHIKV and ZIKV.

DENV infection occurs more frequently in the youngest age groups in Colombia, with the highest incidence being reported in individuals 5 to 14 years of age (15). This epidemiological behavior can be explained by the high endemicity of the country, in which people acquire DENV infection at an early age, generating immunity after the first episode. Similar trends can be expected in the future for CHIKV and ZIKV in several Colombian municipalities because of the similar DENV, ZIKV, and CHIKV transmission cycles that create the endemic establishment of these arboviruses (4). Another characteristic is the higher frequency of cases in men, which may occur because of infection in workplaces where the vector is present.

Campos et al. (7) reported an association of ZIKV infection in 42% of selected patients in northeastern Brazil. The same study also revealed CHIKV co-circulation in 12.5% of investigated cases. Several studies have suggested that the related arboviruses show a level of cross-protection. That is, prior exposure to a virus generates an acquired response after exposure to the second virus, which may decrease the likelihood of sequential infections. The evident reduction in dengue incidence after the co-circulation of the three viruses supports this hypothesis if it occurs in areas endemic for arboviruses. That is because in susceptible persons, no preexisting immune response can be expected for any of the arboviruses, and thus a low cross-response would occur (8, 14).

The data in this study are similar to data reported for the city of São José do Rio Preto, Brazil. In 2016, that city experienced a dengue outbreak characterized by the co-circulation of DENV-1 and DENV-2 and infections with concurrent ZIKV (16). This created epidemiological conditions for the coinfections. In this outbreak, 12 cases of coinfection by DENV and ZIKV were identified.

Related data also come from a cross-sectional study that was conducted in Thailand in 2016 during the rainy season, from May to October (10). The results identified 163 cases in 182 patients (89.56%) infected with DENV, with predominance of DENV-2. Among cases that were positive for DENV, coinfection with CHIKV was identified in 6 patients (3.68%) and coinfection with ZIKV was identified in one patient (0.61%).

In 2014, two patients from New Caledonia were coinfected with DENV and ZIKV (14). Evidence for chikungunya- dengue co-infection has also been found in Angola, Gabon, India, Madagascar, Malaysia, Myanmar, Nigeria, Saint Martin, Singapore, Sri Lanka, Tanzania, Thailand, and Yemen (17). In addition, a case with dengue, chikungunya, and Zika was reported in Colombia (18). The synergistic effects of these viral infections were observed because the patients did not require hospitalization and recovered after mild clinical courses.

Endemic dengue transmission is maintained and persists because of the inadequate and prolonged storage of water for human consumption; misperceptions of individual, collective, and institutional responsibilities for the problem; and noticeable social inequalities (11, 12). Additionally, the interconnection between countries and the higher frequency and intensification of commercial and air transport networks have favored the diffusion, introduction, and transmission of different serotypes because of the rapid transit of individuals with viremia throughout various countries (11, 12).

The same conditions that favor DENV endemicity are likely to contribute to and facilitate the introduction and emergence of ZIKV and CHIKV. For example, there is increased displacement of the population as carriers of viruses move from countries with epidemic transmission to areas that are very receptive to infection because of the persistence of environmental risks and the vulnerability of the entire population to infection (4, 11).

### Limitations

This study has used a secondary source of information, from Colombia’s Public Health Surveillance System. With this surveillance system, there may be underreporting of disease cases and thus underestimation of the disease burden. Additionally, improper classification of a condition may be related to difficulties in obtaining laboratory confirmation of cases. Further research is necessary to understand the current trends in the co-circulation of arboviruses in the countries of Latin America.

### Conclusions and recommendations

In Colombia in 2015, there was a higher incidence of CHIKV than of ZKV and DENV. In 2016, the incidence of ZKV increased, with a subsequent decrease in the frequency of DENV and CHIKV. This situation may reflect a synergistic effect of these viral infections, given that most of the patients reported did not require hospitalization and recovered after a mild clinical course.

To better understand this phenomenon, additional studies should be performed to assess the immunological cross-protection that can develop among the three viruses and the possibility of synergy of the three infections when coinfection appears.

### Author contributions.

Alejandro Rico and Alexandra Porras designed the study, performed the data analysis, and formulated the discussion section of the manuscript. Aileen Chang, Liliana Encinales, and Rebecca Lynch supported the data analysis and the preparation of the discussion section of the manuscript. All the authors reviewed and approved the final version of the manuscript.

### Acknowledgments.

We thank the Instituto Nacional de Salud in Colombia for publishing the data in the epidemiological reports on their website, which we consulted for this study.

### Funding.

The study was supported with resources from the Universidad El Bosque.

### Disclaimer.

The authors hold sole responsibility for the views expressed in the manuscript, which may not necessarily reflect the opinion or policy of the *RPSP/PAJPH* and/or PAHO.
